# Beneficial Effects of a Lutein-Zeaxanthin Complex on Macular Pigment Optical Density Levels of Healthy Individuals With Prolonged Screen Time

**DOI:** 10.7759/cureus.79481

**Published:** 2025-02-22

**Authors:** Vadiraj G Bharadwaj, Thirumalesh MB, Ashwath HV, Anzar CA, Sundaram R, Prasad CP, Joseph MV, Bineesh Eranimose, Prasanna A Reddy

**Affiliations:** 1 Research and Development, Olive Lifesciences Pvt. Ltd., Bangalore, IND; 2 Ophthalmology, Narayana Nethralaya, Bangalore, IND; 3 Clinical Research, SCORES, Bangalore, IND

**Keywords:** blue light effects, contrast sensitivity, heterochromatic flicker photometry, liver function tests, lutein and zeaxanthin, mpod, quality of sleep, renal function tests, screen exposure

## Abstract

Introduction

Macular pigment (MP), consisting of lutein (L) and zeaxanthin (Z), is believed to provide retinal protection against photo-oxidative damage. The objective of the study was to evaluate the effect of lutein and zeaxanthin complex 5:1 (extracted from marigold flowers) supplementation on macular pigment optical density (MPOD), contrast sensitivity, and quality of sleep in healthy subjects who exposed themselves to an electronic gadget screen for a minimum of 8 hours every day. This study also aimed to assess the long-term safety of the supplement by administering it for 8 months in one of the groups. The study also assessed the retention effects of lutein and zeaxanthin on MPOD after discontinuation of supplementation.

Methods

The study was registered with the Clinical Trial Registry of India (CTRI/2022/12/048392). Subjects were screened as per the defined inclusion and exclusion criteria. Subjects aged 18-55 years with a screen time of at least 8 hours daily and MPOD values below 0.8 were recruited. The study, conducted at Narayana Nethralaya Super Specialty Eye Hospital, Bangalore, spanned from December 2022 to May 2024. This was a randomized, placebo-controlled, crossover study. Of the 96 volunteers screened for this study, 71 were recruited, and 60 completed the study. Subjects were divided into 3 groups, viz. A, B, and C. Group A received lutein 10mg and zeaxanthin 2mg twice daily for the first four months, had a wash-off period of 15 days, and continued with the same supplementation for the remaining four months. Group B received lutein 10mg and zeaxanthin 2mg twice daily for the first four months, had a wash-off period of 15 days, and then switched over to a placebo for the next four months. Group C received a Placebo for the first 4 months, had a wash-off period of 15 days, and then switched over to lutein 10mg and zeaxanthin 2mg for the next four months.

All the subjects were given either, lutein and zeaxanthin complex-5:1 or placebo capsules as per the randomization chart prepared computationally. Subjects were analyzed for their MPOD values, contrast sensitivity scores, and quality of sleep. Intraocular pressure, retinal thickness, renal function tests, and liver function tests were conducted during visits to ensure clinical safety.

Results

After supplementation with the lutein-zeaxanthin complex-5:1, the average MPOD (at 1^ᴏ^ eccentricity) increased significantly. At the first visit, the mean MPOD for Groups A, B, and C were 0.3, 0.22, and 0.29 (right eye) and 0.31, 0.27, and 0.27 (left eye), respectively. At the second visit, these values were 0.61, 0.66, and 0.21 (right eye) and 0.54, 0.53, and 0.2 (left eye). By the third visit, the mean MPOD values were 0.7, 0.65, and 0.38 (right eye) and 0.66, 0.53, and 0.36 (left eye). Supplementation significantly improved MPOD, contrast sensitivity, and the quality of sleep compared to placebo.

Conclusions

The supplementation with lutein and zeaxanthin resulted in higher MPOD values as compared to that of the placebo. This intervention also led to improvement in contrast sensitivity and quality of sleep. Lutein and zeaxanthin complex-5:1 may be a promising remedial measure for increasing the MPOD of people exposed to prolonged screen time.

## Introduction

The beneficial carotenoids lutein and zeaxanthin (L and Z) are found in a variety of fruits and vegetables. Lutein and zeaxanthin are also referred to as macular carotenoids because of their preferential concentration in the macula of the human retina [1,2]. Lutein (L) [(3R, 3R, 6R)-lutein] and two zeaxanthin (Z) stereoisomers, (3R, 3R)-zeaxanthin and (3R, 3S; meso)-zeaxanthin (meso-zeaxanthin), are the components of the macular pigment (MP) [3]. The MP filters out blue light and absorbs visible light with a short wavelength. Lutein and zeaxanthin are known to absorb high blue light and protect the retina in the eye; they also possess antioxidant properties. The benefits of lutein and zeaxanthin for skin, cognitive, and ocular health have been well studied. Visual performance is enhanced by lutein and zeaxanthin accumulation in the macula of the human eye. Supplementation with lutein and zeaxanthin has been shown to decrease the risk of age-related macular degeneration (AMD) [4-12]. AMD is primarily caused by choroidal neovascularization (CNV), and lutein supplementation significantly suppresses the inflammatory process associated with CNV in in vivo animal models of AMD [13]. Regular intake of foods rich in carotenoids has been associated with a lower risk of developing a number of chronic conditions, including cardiovascular disorders, cataracts, AMD, and photosensitivity diseases [14,15].

The MP has an antioxidative effect because it suppresses oxygen radicals produced in the photoreceptors and retinal pigment epithelium (RPE) by blue light irradiation [16,17]. Lutein is also known to reduce inflammation [18]. Chronic inflammation and oxidative damage are the main causes of AMD [14,19-23], which is the largest cause of legal blindness in the elderly. Therefore, the MP serves as an important physiological protection against AMD. Previous research showed lower macular pigment optical density (MPOD) in AMD subjects when compared to healthy individuals, and it was hypothesized that lower MPOD could be a risk factor for the development of AMD [24-27].

Genetic and environmental risk factors have been identified for age-related macular degeneration [28,29]. According to the risk factor profile, oxidative stress has been linked to the pathophysiology of the disease associated with low-grade inflammation and hypoxia in the outer retina [30,31]. The concept that low MPOD is a risk factor for the disease is consistent with this view since lutein and zeaxanthin, which are found naturally in the macula, have strong antioxidative qualities [32]. Data from several large-scale studies show a connection between low lutein and zeaxanthin intake and an increased risk of AMD. Major studies on carotenoids include the Age-Related Eye Disease Study (CAREDS), the Blue Mountain Eye Study, and the Age-Related Eye Disease Study (AREDS). In numerous clinical studies, the bulk of which included young subjects, lutein and zeaxanthin administration were reported to improve visual processing speed and reaction times [33,34]. 

The lutein and zeaxanthin complex-5:1 combination is a formulation of *Tagetes erecta* (Marigold) extract that synergistically combines the unique and individual effects of lutein and zeaxanthin. A similar combination of lutein and zeaxanthin has been explored in ARED2. This formulation has both lutein and zeaxanthin in an approximate ratio found in foods, which results in excellent absorption. Lutein and zeaxanthin have been reported to have better absorption profiles in the presence of a lipid media. Sunflower oil is the preferred lipid diluent as it is likely to allow for better absorption of these carotenoids [35].

In this present study, the study volunteers were supplemented with both lutein and zeaxanthin complex-5:1 or a placebo for 8 months to assess their effects on MPOD. Heterochromatic flicker photometry was used to evaluate MPOD and eye stress response by protecting photoreceptor cells from blue light effects.

Objective

This study is an attempt to assess and associate the correlation between MPOD levels of the population with their screen times and improvement of MPOD post-supplementation with lutein and zeaxanthin. The study also aimed to determine the effect of supplementation on contrast sensitivity and quality of sleep.

## Materials and methods

The institutional review board of Narayana Nethralaya Super Specialty Eye Hospital approved this randomized controlled trial vide Letter number C/2022/12/13. This study was performed in accordance with the principles stated in the Declaration of Helsinki and its subsequent amendments and the Good Clinical Practice Guideline. All subjects provided written informed consent for participating in the clinical study as per the protocol. The study was registered with the Clinical Trial Registry of India vide number CTRI/2022/12/048392.

**Figure 1:**
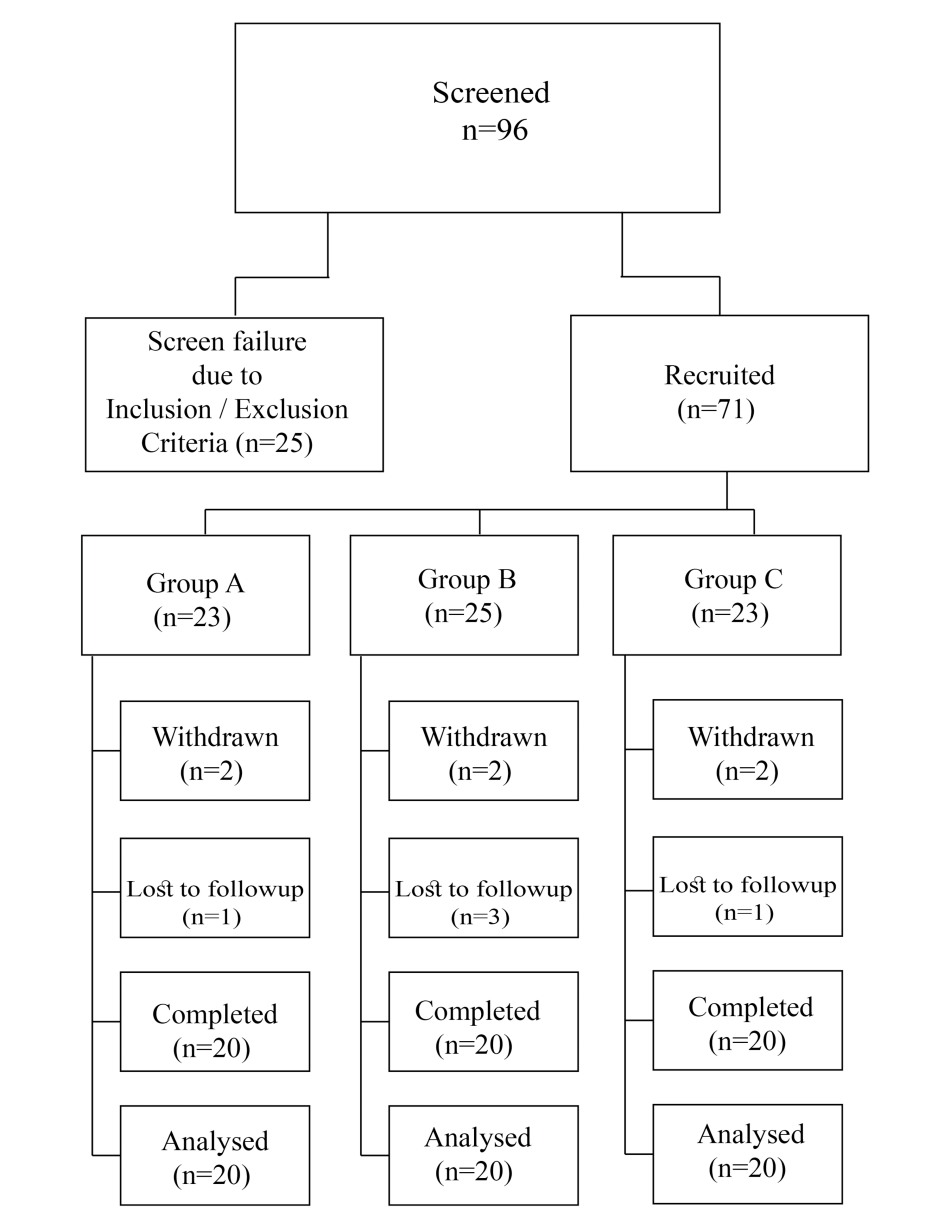
CONSORT diagram

Of the 96 volunteers screened for this study, 71 were recruited, and 60 completed the study. Figure 1 provides the consort diagram describing the number of volunteers screened, recruited, dropped out, and the number of volunteers who completed the study. The study was conducted as a randomized, placebo-controlled, crossover, and double-blind trial at Narayana Nethralaya Super Specialty Eye Hospital Bangalore between December 2022 to May 2024. In this single-center study, 60 healthy subjects of both genders were screened and recruited according to the inclusion and exclusion criteria. The subjects who participated in the current study were healthy except for their MPOD values. Those who agreed to sign the informed consent form were enrolled in the trial, with each subject participating for a duration of 8 months. Subjects received either capsules containing 10 mg of lutein and 2 mg of zeaxanthin, or a placebo. As stipulated by the protocol, 60 subjects were randomly divided into three groups: A, B, and C.

As per Petra et al, the elimination half-life is around 3.26 days [36]. Hence, considering 5 half-life cycles corresponding to approximately 16.3 days, we considered a wash-off period of two weeks for the current study. Twenty subjects in group ‘A’ received MaQxan®-5:1 (lutein and zeaxanthin complex-5:1) (Olive Lifesciences, India) for 8 months with a wash-off period of 15 days after the first 4 months to evaluate the safety of the supplement upon continuous exposure for a longer period of time. While the safety of lutein and zeaxanthin has been well established and reported in multiple studies earlier, the safety of the supplement/formulation MaQxan®-5:1 has been evaluated in this study. Twenty subjects in group ‘B’ received lutein and zeaxanthin complex-5:1 (MaQxan®-5:1) for the first 4 months, had a wash-off period of 15 days, and then received a placebo for the next 4 months. Twenty subjects in group ‘C’ received the placebo for the first 4 months, had a wash-off period of 15 days, and then received the lutein and zeaxanthin complex-5:1 (MaQxan®-5:1) for the next 4 months.

Inclusion criteria

The inclusion criteria included subjects aged between 18 and 55 years, subjects having an MPOD value of less than 0.8, and those having a daily screen time of 8 hours or more, involving activities such as using laptops, mobile phones, or similar devices.

Exclusion criteria

Exclusion criteria included individuals under 18 years of age or over 55 years, enrollment in a similar clinical trial within the past six months, known limitations of care, a known allergy or contraindication to lutein or zeaxanthin, chronic diseases or illnesses that, in the investigator's opinion, could affect the metabolism or absorption of lutein and zeaxanthin, pregnant or actively breastfeeding women, and subjects who were unable or unwilling to provide written informed consent, either themselves or through a legal representative.

Selection of doses and timing of doses in the study

All the subjects were given capsules containing either 10 mg lutein and 2 mg zeaxanthin, or placebo, administered orally twice daily for a period of 8 or 4 months, as per the distribution chart.

Prior and concurrent therapy

Assessment of the safety of the clinical study subjects was conducted through the standardized case reporting forms where the participants list all the supplements or medications (including the over-the-counter medications that may have been used for general sickness like cough, cold, or mild fevers) that they would have taken in the recent past before participating in the trial. To ensure accuracy and completeness, investigators conducted thorough medication reconciliation at study entry and at regular intervals thereafter. Compliance with Good Clinical Practice (GCP) guidelines and local regulatory requirements was maintained throughout this process.

Adherence to treatment

All study treatments were administered by the study investigator or a designated member of staff. Supplement accountability was ensured by the investigator or designated staff keeping precise records of the dates and quantities of supplements received, the individuals to whom they were dispensed, and any supplies that were unintentionally or purposefully destroyed. All of these details were documented on an accountability form. At the end of the trial, all unused clinical supplies and the accountability forms were reconciled.

Efficacy variables 

Macular Pigment Optical Density

The heterochromatic flicker photometry, one of the most reliable and accurate devices for measuring MPOD, was employed to measure the MPOD. The measurement session includes two sets of measurements. Initially, observers are asked to fixate on a central 1° target. The target is composed of two alternating LEDs, blue (465nm) and Green (530nm), with a luminance of up to 200cd/m^2^. They are superimposed on a white light pedestal (colour temperature 5500K). The blue LED is absorbed by macular pigments, while the green LED is absorbed negligibly. On each measurement, the two LEDs start to flicker at a rate of 60Hz, which gradually reduces at a rate of 6 Hz/ second. The flickering rate of 60Hz is above the critical flicker fusion rate; as a result, initially, the target appears static. Observers are asked to press the response button as soon as a flicker is detected. Measurements are obtained for a series of green-blue luminance ratios. The software connected to the instrument gives the MPOD values.

Test for Contrast Sensitivity

**Figure 2:**
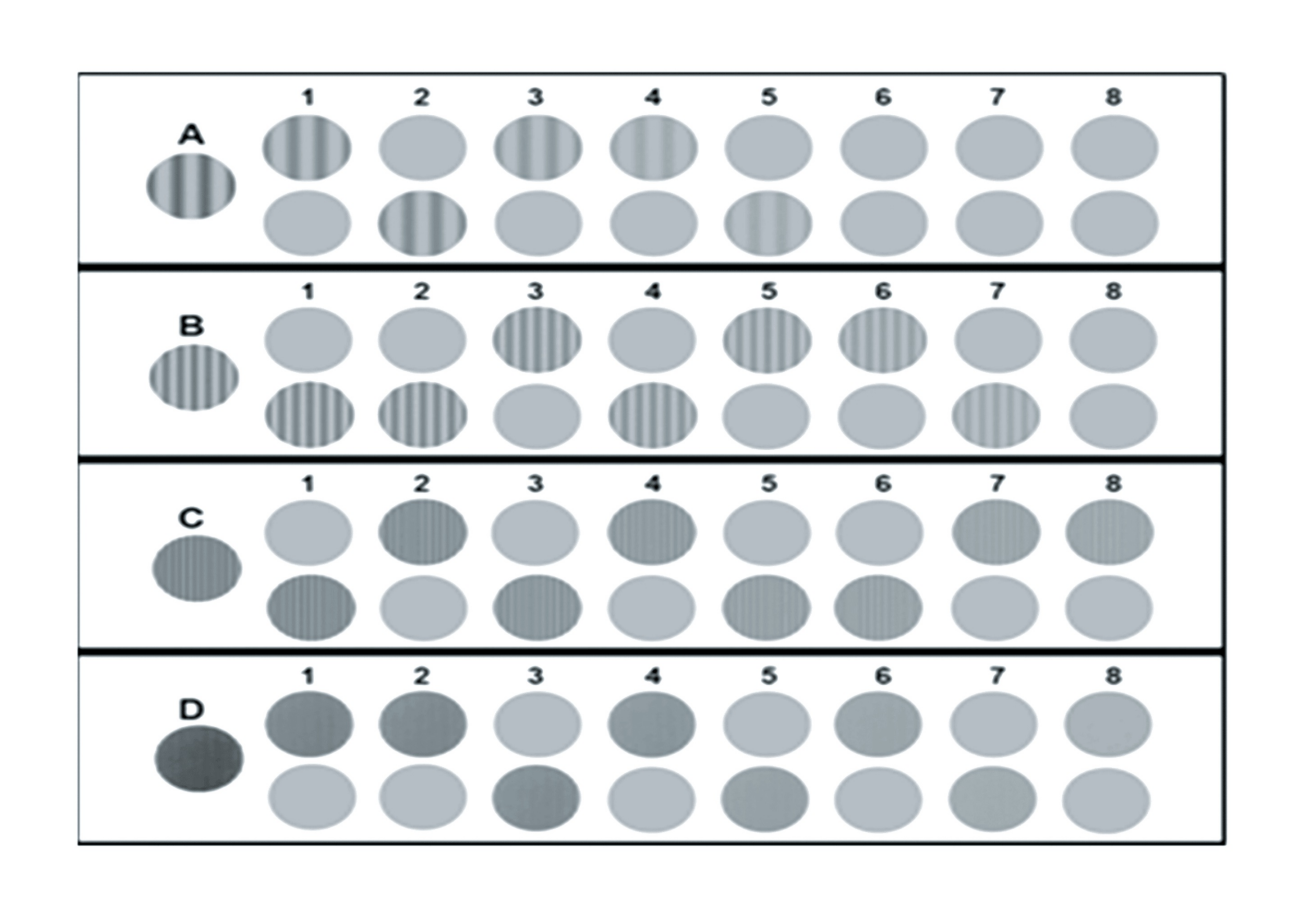
Contrast sensitivity chart A, B, C, and D in the above chart represent sine wave gratings with different spatial frequencies of 3, 6, 12, and 18 cycles/degree (CPD). The numbers 1–8  represent sine wave grating images with changing contrast. The CVS-1000 standardized contrast sensitivity testing instrument (VectorVision, Greenville, Ohio, USA) [37] was used for testing contrast sensitivity. Image used with permission from VectorVision, Greenville, Ohio, USA

The eye contrast sensitivity test measures a subject’s ability to distinguish between progressively finer increments of light versus dark (contrast). This test was important for identifying how much contrast a subject can see comfortably and is often used to evaluate functional visual performance in various conditions, such as cataracts, glaucoma, and macular degeneration. To conduct the test, the subject was seated in a comfortable, well-lit room at about a distance of 2.5 meters away and asked to look at a chart or screen displaying patterns (gratings/stripes) at various levels of contrast. The patterns begin with high contrast and gradually progress to low contrast levels. The subject indicates whether they can distinguish the pattern. The smallest pattern they can detect determines their contrast sensitivity. Each eye is tested separately. Figure 2 shows the chart used to test the contrast sensitivity.

Quality of Sleep Questionnaire

A quality of sleep questionnaire was created based on the experience of the authors. The questionnaire data comprised responses from participants regarding five distinct questions pertaining to the quality of sleep. These responses were structured in an ordinal fashion, where the selected answers dictated the ranking or grade. The order of these levels varied for each question and was determined based on the unique characteristics of each question (Table 1).

**Table 1:** Questions mentioned in sleep questionnaire QOL: Quality of life

Question	Choice that represents the best QOL	Choices that represents intermediate QOL		Choice that represents worst QOL
Q1 (How many hours you sleep in a day)	A (More than 8 hours)	B (Between 6 to 8 hours)	C (Between 4 to 6 hours)	D (Less than 4 hours)
Q2 (How many times in a day you sleep)	A (Only at night)	B (Afternoon and night)	C (In between work)	D (When I get time)
Q3 (How many times you wake up during sleep)	D (Full good sleep)	B (2 to 3 times)	C (At least once)	A (More than 3 times)
Q4 (How do you feel after getting up from sleep)	A (Very relaxed)	B (Relaxed)	C (Disturbed)	D (Very disturbed)
Q5 (How your eyes feel after waking up from sleep)	A (Good)	B (Sleepy)	C (Dry)	D (Irritating)

All subjects were provided with clear instructions on how to fill out the questionnaire. Each participant was given the opportunity to fill out the questionnaire in a quiet, private setting. The questionnaire was answered three times during the study, namely at the time of screening, the 4th month, and at the end of the study. Sessions were held at consistent times according to protocol to avoid variability in sleep patterns. Questionnaires were collected promptly after completion and regular checks were done to ensure data accuracy and completeness.

Quality control and monitoring

All study personnel were trained on the protocol, Good Clinical Practice (GCP), and data handling procedures. The trial's conduct and the data accuracy were reviewed periodically while the study was being observed. Implemented QC checks at various stages of data collection and entry to identify and rectify errors.

Safety and reporting of adverse events

Reporting of adverse events was encouraged and analyzed to detect any safety concerns. Timely and accurate reporting of any adverse events was done to maintain data integrity. HbA1c, serum creatinine, urine, sodium, potassium, chloride, urea, bilirubin total, bilirubin direct, bilirubin indirect, ALP (alkaline phosphatase), ALT (alanine transaminase), AST (aspartate aminotransferase), GGT (gamma-glutamyl transferase), TT3 (total triiodothyronine), TT4 (total thyroxine), TSH (thyroid-stimulating hormone) IOP (Intraocular pressure) and retinal thickness were measured during every visit to evaluate the safety of subjects during the study duration.

Randomization and blinding

The 3x3 block randomization method was used. Randomization was done computationally. Subjects were allocated to groups according to a randomized sequence in each block. Supplement and placebo capsules were of similar size, colour, and shape to maintain the blinding. The capsules were packed in identical containers. All containers were sequentially numbered and randomized using a computer-generated numbering which was maintained by a designated staff. Investigator and subjects were blinded.

Descriptive statistics 

Sample Size Calculation

Considering an average South Indian population (as the trial was conducted in the southern part of India and the subjects who participated in the study were south Indians), an MPOD value of 0.37 with a standard deviation of 0.17, and expecting a twofold increase in MPOD following the treatment, we calculated the sample size required to achieve more than 90% power [38]. Our calculations indicated that the required sample size is approximately 6 assuming a two-sided alternative hypothesis for a paired t-test. The calculation was done using the "pwr" Package in R (R package version 1.3-0). However, we proceeded ahead with 20 subjects per group.

Analysis of Statistical Significance

Descriptive statistical analysis was conducted using the R package (https://www.r-project.org/). The data comprised both numeric and categorical variables, each of which was processed accordingly: averages were computed for numeric variables, while counts were determined for categorical variables. The analyst who interpreted the data was blinded to the treatment groups to avoid bias in data analysis and interpretation. The analysis was conducted using the following statistical methods

For comparing the baseline values (i.e., visit-1 for Groups A, B, and C), a one-way analysis of variance (ANOVA) was used. Further, pairwise t-tests with Bonferroni alpha correction were used to compare the numeric values across the three visits following the treatment. This approach was chosen for several reasons. Firstly, pairwise t-tests allow for the comparison of means between two groups, making them suitable for analyzing the differences in numeric values observed at different time points within the same subjects. Additionally, applying Bonferroni correction helps mitigate the issue of multiple comparisons by adjusting the significance level to maintain an overall alpha level, thus reducing the risk of type I errors. ANOVA was used to analyze the difference between the means of more than two groups. The Kruskal-Wallis test was used for assessing differences between groups. The chi-square test was used to determine a significant relationship between two categorical variables. The Wilcoxon signed-rank test was used to assess the magnitude of the difference between groups using two matched samples. All the statistical data are expressed as Mean ± SD.

## Results

Subject characteristics

Of the 96 volunteers screened for this study, 71 were recruited, and 60 completed the study. Six participants withdrew from the study as they had to relocate and 5 participants were lost to follow-up. No protocol deviations were observed with the participants who completed the study process.

Table 2 shows the demographic data of the subjects who participated in the study. Table 3 in specific shows the exposure of subjects to different types of screens and electronic gadgets

**Table 2:** Subject characteristic with respect to age, gender and body features Group A received MaQxan®-5:1 (lutein and zeaxanthin complex-5:1) for complete 8 months with a wash-off period of 15 days after the first 4 months. Group B received MaQxan®-5:1 (lutein and zeaxanthin complex-5:1) for the first 4 months, had a wash-off period of 15 days, and then received a placebo for the next 4 months. Group C placebo for the first 4 months, had a wash-off period of 15 days, and then received the MaQxan®-5:1 (lutein and zeaxanthin complex-5:1) for the next 4 months.

Parameter		Group A (N=20)	Group B (N=20)	Group C (N=20)	Total (N=60)
Gender	Male	11 (55.0%)	11 (55.0%)	6 (30.0%)	28 (46.7%)
	Female	9 (45.0%)	9 (45.0%)	14 (70.0%)	32 (53.3%)
Age (years)	Mean	37.7 ± 13.2	39.1 ± 11.4	34.5 ± 11.2	37.1 ± 11.9
	Min	19	18	21	-
	Max	55	55	53	-
Height (cm)	Mean	161 ± 10.4	163 ± 11.3	165 ± 10.2	163 ± 10.5
	Min	144	143	147	-
	Max	180	189	183	-
Weight (kg)	Mean	67.3 ± 16.7	67.8 ± 10.3	64.6 ± 10.0	66.6 ± 12.6
	Min	47	47	47	-
	Max	114	90	86	-

**Table 3:** Usage of electronic gadgets Group A received MaQxan®-5:1 (lutein and zeaxanthin complex-5:1) for complete 8 months with a wash-off period of 15 days after the first 4 months. Group B received MaQxan®-5:1 (lutein and zeaxanthin complex-5:1) for the first 4 months, had a wash-off period of 15 days, and then received a placebo for the next 4 months. Group C placebo for the first 4 months, had a wash-off period of 15 days, and then received the MaQxan®-5:1 (lutein and zeaxanthin complex-5:1) for the next 4 months.

Parameter	Group A	Group B	Group C	Total
Mobile				
Yes	20 (100%)	20 (100%)	20 (100%)	60 (100%)
Desktop				
No	12 (60.0%)	15 (75.0%)	14 (70.0%)	41 (68.3%)
Yes	8 (40.0%)	5 (25.0%)	6 (30.0%)	19 (31.7%)
Laptop				
No	15 (75.0%)	14 (70.0%)	12 (60.0%)	41 (68.3%)
Yes	5 (25.0%)	6 (30.0%)	8 (40.0%)	19 (31.7%)
Television				
No	7 (35.0%)	4 (20.0%)	9 (45.0%)	20 (33.3%)
Yes	13 (65.0%)	16 (80.0%)	11 (55.0%)	40 (66.7%)

Macular pigment optical density analysis

Baseline Characteristics Before the Treatment

As shown in Table 4, the baseline values of MPOD were comparable across different groups and the differences were not statistically significant.

**Table 4:** Mean MPOD values across visits Group A received MaQxan®-5:1 (lutein and zeaxanthin complex-5:1) for complete 8 months with a wash-off period of 15 days after the first 4 months. Group B received MaQxan®-5:1 (lutein and zeaxanthin complex-5:1) for the first 4 months, had a wash-off period of 15 days, and then received a placebo for the next 4 months. Group C placebo for the first 4 months, had a wash-off period of 15 days, and then received the MaQxan®-5:1 (lutein and zeaxanthin complex-5:1) for the next 4 months. MPOD : Macular Pigment Optical Density * Significant difference between Visit 2 to Visit 1 @ Significant difference between Visit 3 to Visit 1 # Significant difference between Visit 3 to Visit 2 NS : Not Significant Statistical significance measure by Pairwise t-test with Bonferroni correction

	Visit	MPOD-RIGHT	t-values and p-values for right eyes	MPOD-LEFT	t-values and p-values for left eyes
Mean ± SD MPOD values (1° eccentricity) of Group-A	Visit 1	0.3 ± 0.17	Baseline	0.31 ± 0.17	Baseline
	Visit 2	0.61 ± 0.4^*^	t=4.14, p=0.0016	0.54 ± 0.34^*^	t=3.69, p<0.005
	Visit 3	0.7 ± 0.36^@^	t=5.43, p=0.0001	0.66 ± 0.32^@#^	t=5.64, p<0.001^@^; t=2.92, p=0.026^#^
Mean ± SD MPOD values (1° eccentricity) of Group-B	Visit 1	0.22 ± 0.18	Baseline	0.27 ± 0.2	Baseline
	Visit 2	0.66 ± 0.34^*^	t=5.44, p=0.000089	0.53 ± 0.29^*^	t=5.30, p<0.001
	Visit 3	0.65 ± 0.34^@^	t=5.30, p=0.000121	0.53 ± 0.28^@^	t=5.24, p<0.001
Mean ± SD MPOD values (1° eccentricity) of Group-C	Visit 1	0.29 ± 0.2	Baseline	0.27 ± 0.18	Baseline
	Visit 2	0.21 ± 0.25	NS	0.2 ± 0.24	NS
	Visit 3	0.38 ± 0.2^#^	t=6.001, p=0.000026	0.36 ± 0.25^#^	t=-3.90, p<0.003

Comparison of MPOD Values Between Different Visits for Group A

The data in Table 4 show a significant increase in mean MPOD-RIGHT values across visits. At visit 1, the mean was 0.3, which rose to 0.6 at visit 2 (a twofold increase) and to 0.7 at visit 3 (a 2.3-fold increase). The increase in MPOD-RIGHT values at visit-2 and visit-3 compared to visit 1 was statistically significant (p<0.05, pairwise t-test with Bonferroni correction). Although the MPOD-RIGHT value was slightly higher at visit 3 than at visit 2, this difference was not statistically significant (p=0.08). These results suggest that the Lutein and Zeaxanthin complex-5:1 treatment had a positive effect on increasing MPOD.

Further analysis of MPOD distribution across visits is presented in Table 5. At visit 1, 50% of participants had MPOD-RIGHT values below 0.325, while at visit 2 and visit 3, the median values increased to 0.54 and 1.0, respectively. Similarly, the median MPOD-LEFT values also increased from visit 1 to visit 2 and visit 3. The maximum MPOD values for both eyes reached 1.0, further indicating the positive impact of the treatment.

**Table 5:** Comparison of MPOD scores between different visits of Group A (percentile values) Group A received MaQxan®-5:1 (lutein and zeaxanthin complex-5:1) for complete 8 months with a wash-off period of 15 days after the first 4 months.

	Percentile	MPOD-LEFT	MPOD-RIGHT
Visit 1	Minimum	0.07	0
	25%	0.195	0.235
	50%	0.29	0.325
	75%	0.46	0.3875
	Maximum	0.62	0.6
Visit 2	Minimum	0	0
	25%	0.325	0.31
	50%	0.48	0.54
	75%	1	1
	Maximum	1	1
Visit 3	Minimum	0.17	0.02
	25%	0.3775	0.3475
	50%	0.585	1
	75%	1	1
	Maximum	1	1

Comparison of MPOD Values Between Different Visits for Group B

In Group B, the mean MPOD-RIGHT values increased nearly threefold from visit 1 to visit 2, and retained similar values in visit 3. A similar trend was observed for MPOD-LEFT, which rose from 0.27 to 0.53 between visit 1 and visit 2, and this increase was sustained through visit 3. The increase in MPOD values from visit 1 to visit 2, as well as from visit 1 to visit 3, was statistically significant (p<0.05, pairwise t-test with Bonferroni correction), indicating a positive effect of the lutein and zeaxanthin complex-5:1 treatment in increasing MPOD.

Table 6 shows that the median MPOD-RIGHT and MPOD-LEFT values increased at visits 2 and 3 compared to visit 1. At visit 1, 50% of participants had MPOD-RIGHT and MPOD-LEFT values below 0.26 and 0.23, respectively. By visit 2, more than 50% had values greater than 0.495 for MPOD-RIGHT and 0.61 for MPOD-LEFT. The maximum MPOD value for both eyes reached 1.0 at visit 2 and remained at this level through visit 3.

**Table 6:** Comparison of MPOD scores between different visits of Group B (percentile values) Group B received MaQxan®-5:1 (lutein and zeaxanthin complex-5:1) for the first 4 months, had a wash-off period of 15 days, and then received a placebo for the next 4 months.

	Percentile	MPOD-LEFT	MPOD-RIGHT
Visit 1	Minimum	0	0
	25%	0.095	0.0575
	50%	0.26	0.23
	75%	0.385	0.345
	Maximum	0.6	0.53
Visit 2	Minimum	0.02	0.12
	25%	0.37	0.31
	50%	0.495	0.61
	75%	0.605	1
	Maximum	1	1
Visit 3	Minimum	0	0.12
	25%	0.385	0.3075
	50%	0.515	0.62
	75%	0.6	1
	Maximum	1	1

Comparison of MPOD Values Between Different Visits for Group C

In Group C, the average MPOD-RIGHT values at visits 1 and 2 were 0.29 and 0.21, respectively, with no statistically significant difference between them (p > 0.05, pairwise t-test with Bonferroni correction). However, the MPOD-RIGHT value increased to 0.38 at visit 3, which was a statistically significant increase (p<0.05, pairwise t-test with Bonferroni correction).

A similar trend was observed for MPOD-LEFT, with a significant improvement from visit 2 to visit 3 (p<0.05, pairwise t-test with Bonferroni correction). Additionally, compared to visits 1 and 2, the MPOD values for the 75th percentile were higher during visit 3, as seen in Table 7.

**Table 7:** Comparison of MPOD scores between different visits of Group C (percentile values) Group C placebo for the first 4 months, had a wash-off period of 15 days, and then received the MaQxan®-5:1 (lutein and zeaxanthin complex-5:1) for the next 4 months.

	Percentile	MPOD-LEFT	MPOD-RIGHT
Visit 1	Minimum	0	0
	25%	0.1225	0.1075
	50%	0.36	0.265
	75%	0.41	0.4225
	Maximum	0.55	0.6
Visit 2	Minimum	0	0
	25%	0	0.02
	50%	0.22	0.17
	75%	0.36	0.335
	Maximum	1	1
Visit 3	Minimum	0.02	0.07
	25%	0.1925	0.3
	50%	0.34	0.36
	75%	0.43	0.4625
	Maximum	1	1

Contrast Sensitivity 

Comparison of Contrast Sensitivity Scores Between Different Visits for Group A

Table 8 provides a summary of the number of people and their respective contrast sensitivity scores during different visits for Group A. Compared to visit 1, the number of individuals scoring high on contrast sensitivity parameters increases during visits 2 and 3. The number of individuals scoring 6 was zero during visit 1. When compared to this, during visit 2, 10% of them scored 7. This was maintained during visit 3. For the parameter RC, the number of individuals scoring 8 doubled during visits 2 and 3 compared to visit 1. Similarly, the number of individuals scoring higher in contrast sensitivity increased for other parameters as well (Table 8). Statistical analysis (Wilcoxon signed rank test) indicated that when compared between visits 2 and 1, the increase in contrast sensitivity scores for all the parameters was statistically significant (p<0.05, Wilcoxon signed-rank test). When compared between visit 3 and visit 1, the increase in contrast sensitivity scores for all the parameters was statistically significant (p<0.05, Wilcoxon signed-rank test). When compared between visits 3 and 2, the increase in contrast sensitivity scores for RA, RB, LA, and LB parameters was statistically significant (p<0.05, Wilcoxon signed-rank test). This might indicate a sustained effect of lutein and zeaxanthin complex-5:1 treatment during different visits.

**Table 8:** Comparison of contrast sensitivity scores across visits for subjects in Group A Group A received MaQxan®-5:1 (lutein and zeaxanthin complex-5:1) for complete 8 months with a wash-off period of 15 days after the first 4 months. Right A, B, C, and D or Left A, B, C, and D represent contrast sensitivity scores of the right or left eye for different spatial frequencies of A, B, C, and D corresponding to 3, 6, 12 and 18 cycles/degree (CPD) respectively. The numbers 1-8 represent sine wave grating image with changing contrast. Significance measured by Wilcoxon signed-rank test. LD is not significant for comparison between visit 2 to visit 1 and comparison between visit 3 to visit 2. RC, RD, and LC are not significant (NS) for comparison between visit 3 to visit 2.

Group A	Visit 1	Visit 2	Visit 3	Overall	Test statistics and p-value		
	(N=20)	(N=20)	(N=20)	(N=60)	Visit 2 versus Visit 1	Visit 3 versus Visit 1	Visit 3 versus Visit 2
Right A							
1	2 (10.0%)	0 (0%)	0 (0%)	2 (3.3%)	W=137, p<0.001	W=136, p<0.001	W=127.5, p<0.001
2	0 (0%)	0 (0%)	0 (0%)	0 (0%)			
3	2 (10.0%)	0 (0%)	0 (0%)	2 (3.3%)			
4	11 (55.0%)	6 (30.0%)	2 (10.0%)	19 (31.7%)			
5	5 (25.0%)	12 (60.0%)	7 (35.0%)	24 (40.0%)			
6	0 (0%)	2 (10.0%)	10 (50.0%)	12 (20.0%)			
7	0 (0%)	0 (0%)	1 (5.0%)	1 (1.7%)			
8	0 (0%)	0 (0%)	0 (0%)	0 (0%)			
Right B							
1	0 (0%)	0 (0%)	0 (0%)	0 (0%)	W=93, p=0.004	W=149.5, p<0.001	W=126, p=0.004
2	0 (0%)	0 (0%)	0 (0%)	0 (0%)			
3	2 (10.0%)	0 (0%)	0 (0%)	2 (3.3%)			
4	2 (10.0%)	1 (5.0%)	0 (0%)	3 (5.0%)			
5	6 (30.0%)	3 (15.0%)	1 (5.0%)	10 (16.7%)			
6	7 (35.0%)	8 (40.0%)	7 (35.0%)	22 (36.7%)			
7	3 (15.0%)	8 (40.0%)	9 (45.0%)	20 (33.3%)			
8	0 (0%)	0 (0%)	3 (15.0%)	3 (5.0%)			
Right C							
1	0 (0%)	0 (0%)	0 (0%)	0 (0%)	W=93, p=0.004	W=94, p=0.004	NS
2	0 (0%)	0 (0%)	0 (0%)	0 (0%)			
3	0 (0%)	0 (0%)	0 (0%)	0 (0%)			
4	0 (0%)	0 (0%)	0 (0%)	0 (0%)			
5	1 (5.0%)	0 (0%)	0 (0%)	1 (1.7%)			
6	4 (20.0%)	0 (0%)	0 (0%)	4 (6.7%)			
7	7 (35.0%)	4 (20.0%)	4 (20.0%)	15 (25.0%)			
8	8 (40.0%)	16 (80.0%)	16 (80.0%)	40 (66.7%)			
Right D							
1	0 (0%)	0 (0%)	0 (0%)	0 (0%)	W=21, p=0.017	W=28, p=0.01	NS
2	0 (0%)	0 (0%)	0 (0%)	0 (0%)			
3	0 (0%)	0 (0%)	0 (0%)	0 (0%)			
4	0 (0%)	0 (0%)	0 (0%)	0 (0%)			
5	1 (5.0%)	0 (0%)	0 (0%)	1 (1.7%)			
6	2 (10.0%)	0 (0%)	0 (0%)	2 (3.3%)			
7	4 (20.0%)	1 (5.0%)	0 (0%)	5 (8.3%)			
8	13 (65.0%)	19 (95.0%)	20 (100%)	52 (86.7%)			
Left A							
1	1 (5.0%)	0 (0%)	0 (0%)	1 (1.7%)	W=107, p=0.002	W=153, p<0.001	W=128, p<0.001
2	0 (0%)	0 (0%)	0 (0%)	0 (0%)			
3	3 (15.0%)	0 (0%)	0 (0%)	3 (5.0%)			
4	8 (40.0%)	6 (30.0%)	2 (10.0%)	16 (26.7%)			
5	8 (40.0%)	10 (50.0%)	5 (25.0%)	23 (38.3%)			
6	0 (0%)	4 (20.0%)	11 (55.0%)	15 (25.0%)			
7	0 (0%)	0 (0%)	2 (10.0%)	2 (3.3%)			
Left B							
1	0 (0%)	0 (0%)	0 (0%)	0 (0%)	W=115, p=0.005	W=132.5, p<0.001	W=105.5, p<0.019
2	0 (0%)	0 (0%)	0 (0%)	0 (0%)			
3	0 (0%)	0 (0%)	0 (0%)	0 (0%)			
4	3 (15.0%)	0 (0%)	0 (0%)	3 (5.0%)			
5	6 (30.0%)	4 (20.0%)	1 (5.0%)	11 (18.3%)			
6	7 (35.0%)	7 (35.0%)	5 (25.0%)	19 (31.7%)			
7	4 (20.0%)	8 (40.0%)	11 (55.0%)	23 (38.3%)			
8	0 (0%)	1 (5.0%)	3 (15.0%)	4 (6.7%)			
Left C							
1	0 (0%)	0 (0%)	0 (0%)	0 (0%)	W=113.5, p<0.001	W=108, p=0.002	NS
2	0 (0%)	0 (0%)	0 (0%)	0 (0%)			
3	0 (0%)	0 (0%)	0 (0%)	0 (0%)			
4	0 (0%)	0 (0%)	0 (0%)	0 (0%)			
5	2 (10.0%)	0 (0%)	0 (0%)	2 (3.3%)			
6	3 (15.0%)	0 (0%)	1 (5.0%)	4 (6.7%)			
7	9 (45.0%)	3 (15.0%)	3 (15.0%)	15 (25.0%)			
8	6 (30.0%)	17 (85.0%)	16 (80.0%)	39 (65.0%)			
Left D							
1	0 (0%)	0 (0%)	0 (0%)	0 (0%)	W=15, p=0.027	NS	NS
2	0 (0%)	0 (0%)	0 (0%)	0 (0%)			
3	0 (0%)	0 (0%)	0 (0%)	0 (0%)			
4	0 (0%)	0 (0%)	0 (0%)	0 (0%)			
5	0 (0%)	0 (0%)	0 (0%)	0 (0%)			
6	2 (10.0%)	0 (0%)	0 (0%)	2 (3.3%)			
7	3 (15.0%)	1 (5.0%)	0 (0%)	4 (6.7%)			
8	15 (75.0%)	19 (95.0%)	20 (100%)	54 (90.0%)			

Comparison of Contrast Sensitivity Scores Between Different Visits for Group B

Table 9 provides a summary of the number of people and their respective contrast sensitivity scores during different visits for Group B. Compared to visit 1, the number of individuals scoring high on contrast sensitivity parameters increased during visit 2. For the parameter RA, the number of individuals scoring 6 increased to 25% as compared to visit 1. Similarly, for RB, the number of individuals scoring 7 almost doubled during visit 2. For RC, 95% of the individuals scored 8 during visit 2, whereas during visit 1, only 25% of the individuals scored 8. For RD and LA, similar trends were observed.

**Table 9:** Comparison of contrast sensitivity scores across visits for subjects in Group B Group B received MaQxan®-5:1 (lutein and zeaxanthin complex-5:1) for the first 4 months, had a wash-off period of 15 days, and then received a placebo for the next 4 months. Right A, B, C, and D or Left A, B, C, and D represent contrast sensitivity scores of the right or left eye for different spatial frequencies of A, B, C, and D corresponding to 3, 6, 12 and 18 cycles/degree (CPD) respectively. The numbers 1-8 represent sine wave grating images with changing contrast. Significance was measured using the Wilcoxon signed-rank test. NS: not significant

Group B	Visit 1	Visit 2	Visit 3	Overall	Test statistics and p-value		
	(N=20)	(N=20)	(N=20)	(N=60)	Visit 2 versus Visit 1	Visit 3 versus Visit 1	Visit 3 versus Visit 2
Right A							
1	0 (0%)	0 (0%)	0 (0%)	0 (0%)	W=72.5, p=0.003	W=99, p=0.001	NS
2	0 (0%)	0 (0%)	0 (0%)	0 (0%)			
3	2 (10.0%)	0 (0%)	0 (0%)	2 (3.3%)			
4	11 (55.0%)	8 (40.0%)	5 (25.0%)	24 (40.0%)			
5	6 (30.0%)	6 (30.0%)	9 (45.0%)	21 (35.0%)			
6	0 (0%)	5 (25.0%)	5 (25.0%)	10 (16.7%)			
7	1 (5.0%)	1 (5.0%)	1 (5.0%)	3 (5.0%)			
8	0 (0%)	0 (0%)	0 (0%)	0 (0%)			
Right B							
1	0 (0%)	0 (0%)	0 (0%)	0 (0%)	W=128.5, p<0.001	W=99, p=0.001	NS
2	0 (0%)	0 (0%)	0 (0%)	0 (0%)			
3	0 (0%)	0 (0%)	0 (0%)	0 (0%)			
4	0 (0%)	0 (0%)	0 (0%)	0 (0%)			
5	6 (30.0%)	1 (5.0%)	1 (5.0%)	8 (13.3%)			
6	9 (45.0%)	6 (30.0%)	7 (35.0%)	22 (36.7%)			
7	5 (25.0%)	10 (50.0%)	9 (45.0%)	24 (40.0%)			
8	0 (0%)	3 (15.0%)	3 (15.0%)	6 (10.0%)			
Right C							
1	0 (0%)	0 (0%)	0 (0%)	0 (0%)	W=120, p<0.001	W=105, p<0.001	NS
2	0 (0%)	0 (0%)	0 (0%)	0 (0%)			
3	0 (0%)	0 (0%)	0 (0%)	0 (0%)			
4	0 (0%)	0 (0%)	0 (0%)	0 (0%)			
5	2 (10.0%)	0 (0%)	0 (0%)	2 (3.3%)			
6	1 (5.0%)	0 (0%)	0 (0%)	1 (1.7%)			
7	12 (60.0%)	1 (5.0%)	2 (10.0%)	15 (25.0%)			
8	5 (25.0%)	19 (95.0%)	18 (90.0%)	42 (70.0%)			
Right D							
1	0 (0%)	0 (0%)	0 (0%)	0 (0%)	W=10, p=0.045	W=10, p=0.045	NS
2	0 (0%)	0 (0%)	0 (0%)	0 (0%)			
3	0 (0%)	0 (0%)	0 (0%)	0 (0%)			
4	0 (0%)	0 (0%)	0 (0%)	0 (0%)			
5	1 (5.0%)	0 (0%)	0 (0%)	1 (1.7%)			
6	0 (0%)	0 (0%)	0 (0%)	0 (0%)			
7	3 (15.0%)	0 (0%)	0 (0%)	3 (5.0%)			
8	16 (80.0%)	20 (100%)	20 (100%)	56 (93.3%)			
Left A							
1	0 (0%)	0 (0%)	0 (0%)	0 (0%)	W=72, p=0.003	W=98, p=0.001	NS
2	0 (0%)	0 (0%)	0 (0%)	0 (0%)			
3	1 (5.0%)	0 (0%)	0 (0%)	1 (1.7%)			
4	10 (50.0%)	7 (35.0%)	5 (25.0%)	22 (36.7%)			
5	9 (45.0%)	7 (35.0%)	9 (45.0%)	25 (41.7%)			
6	0 (0%)	6 (30.0%)	6 (30.0%)	12 (20.0%)			
7	0 (0%)	0 (0%)	0 (0%)	0 (0%)			
8	0 (0%)	0 (0%)	0 (0%)	0 (0%)			
Left B							
1	0 (0%)	0 (0%)	0 (0%)	0 (0%)	W=98.5, p<0.001	W=115, p=0.005	NS
2	0 (0%)	0 (0%)	0 (0%)	0 (0%)			
3	0 (0%)	0 (0%)	0 (0%)	0 (0%)			
4	0 (0%)	0 (0%)	0 (0%)	0 (0%)			
5	7 (35.0%)	1 (5.0%)	1 (5.0%)	9 (15.0%)			
6	7 (35.0%)	7 (35.0%)	8 (40.0%)	22 (36.7%)			
7	6 (30.0%)	10 (50.0%)	9 (45.0%)	25 (41.7%)			
8	0 (0%)	2 (10.0%)	2 (10.0%)	4 (6.7%)			
Left C							
1	0 (0%)	0 (0%)	0 (0%)	0 (0%)	W=91, p<0.001	W=78, p<0.001	NS
2	0 (0%)	0 (0%)	0 (0%)	0 (0%)			
3	0 (0%)	0 (0%)	0 (0%)	0 (0%)			
4	0 (0%)	0 (0%)	0 (0%)	0 (0%)			
5	0 (0%)	0 (0%)	0 (0%)	0 (0%)			
6	1 (5.0%)	0 (0%)	0 (0%)	1 (1.7%)			
7	13 (65.0%)	2 (10.0%)	2 (10.0%)	17 (28.3%)			
8	6 (30.0%)	18 (90.0%)	18 (90.0%)	42 (70.0%)			
Left D							
1	0 (0%)	0 (0%)	0 (0%)	0 (0%)	NS	NS	NS
2	0 (0%)	0 (0%)	0 (0%)	0 (0%)			
3	0 (0%)	0 (0%)	0 (0%)	0 (0%)			
4	0 (0%)	0 (0%)	0 (0%)	0 (0%)			
5	0 (0%)	0 (0%)	0 (0%)	0 (0%)			
6	0 (0%)	0 (0%)	0 (0%)	0 (0%)			
7	1 (5.0%)	0 (0%)	0 (0%)	1 (1.7%)			
8	19 (95.0%)	20 (100%)	20 (100%)	59 (98.3%)			

Statistical analysis indicated that, when compared between visit 2 and visit 1, the increase in the contrast sensitivity scores for all the parameters was statistically significant (p<0.05, Wilcoxon signed-rank test). When compared between visit 3 and visit 1, the increase in contrast sensitivity scores for all the parameters was statistically significant (p<0.05, Wilcoxon signed-rank test) indicating that the effect of lutein and zeaxanthin complex-5:1 treatment is maintained even during visit 3.

Comparison of Contrast Sensitivity Scores Between Different Visits for Group C

Table 10 provides a summary of the number of people and their respective contrast sensitivity scores during different visits for Group C. Compared to visit 1 or visit 2, the number of individuals scoring high on contrast sensitivity parameters increased during visit 3. For the parameter RA, the number of individuals scoring 6 increased from 0 to 30% as compared to visit 1 or visit 2. Similarly, for RB, the number of individuals scoring 8 increased to 25% during visit 3, whereas during visit 1 or visit 2, this was zero (no individuals scored 6 during visit 1 or visit 2). Similarly, for other parameters, the number of individuals scoring high in the contrast sensitivity test was higher during visit 3 compared to visit 1.

**Table 10:** Comparison of contrast sensitivity scores across visits for subjects in Group C Group C placebo for the first 4 months, had a wash-off period of 15 days, and then received the MaQxan®-5:1 (lutein and zeaxanthin complex-5:1) for the next 4 months. Right A, B, C and D or Left A, B, C and D represent contrast sensitivity scores of right or left eye for different spatial frequencies of A, B, C, and D corresponding to 3, 6, 12, and 18 cycles/degree (CPD) respectively. The numbers 1-8 represent sine wave grating image with changing contrast. RA, RD, and LD are statistically not significant (NS) for comparison between visit 2 to visit 1. Significance was measured using Wilcoxon signed-rank test.

Group C	Visit 1	Visit 2	Visit 3	Overall	Test statistics and p-value		
	(N=20)	(N=20)	(N=20)	(N=60)	Visit 2 versus Visit 1	Visit 3 versus Visit 1	Visit 3 versus Visit 2
Right A							
1	0 (0%)	0 (0%)	0 (0%)	0 (0%)	NS	W=171, p<0.001	W=190, p<0.001
2	0 (0%)	0 (0%)	0 (0%)	0 (0%)			
3	0 (0%)	0 (0%)	0 (0%)	0 (0%)			
4	13 (65.0%)	13 (65.0%)	0 (0%)	26 (43.3%)			
5	7 (35.0%)	7 (35.0%)	14 (70.0%)	28 (46.7%)			
6	0 (0%)	0 (0%)	6 (30.0%)	6 (10.0%)			
7	0 (0%)	0 (0%)	0 (0%)	0 (0%)			
8	0 (0%)	0 (0%)	0 (0%)	0 (0%)			
Right B							
1	0 (0%)	0 (0%)	0 (0%)	0 (0%)	W=15, p=0.02385	W=153, p<0.001	W=112, p<0.001
2	0 (0%)	0 (0%)	0 (0%)	0 (0%)			
3	0 (0%)	0 (0%)	0 (0%)	0 (0%)			
4	0 (0%)	0 (0%)	0 (0%)	0 (0%)			
5	7 (35.0%)	6 (30.0%)	1 (5.0%)	14 (23.3%)			
6	9 (45.0%)	5 (25.0%)	7 (35.0%)	21 (35.0%)			
7	4 (20.0%)	9 (45.0%)	7 (35.0%)	20 (33.3%)			
8	0 (0%)	0 (0%)	5 (25.0%)	5 (8.3%)			
Right C							
1	0 (0%)	0 (0%)	0 (0%)	0 (0%)	W=24, p=0.036	W=78, p<0.001	W=40, p=0.0112
2	0 (0%)	0 (0%)	0 (0%)	0 (0%)			
3	0 (0%)	0 (0%)	0 (0%)	0 (0%)			
4	0 (0%)	0 (0%)	0 (0%)	0 (0%)			
5	0 (0%)	0 (0%)	0 (0%)	0 (0%)			
6	3 (15.0%)	3 (15.0%)	0 (0%)	6 (10.0%)			
7	11 (55.0%)	6 (30.0%)	5 (25.0%)	22 (36.7%)			
8	6 (30.0%)	11 (55.0%)	15 (75.0%)	32 (53.3%)			
Right D							
1	0 (0%)	0 (0%)	0 (0%)	0 (0%)	NS	W=21, p=0.01	W=15, p=0.018
2	0 (0%)	0 (0%)	0 (0%)	0 (0%)			
3	0 (0%)	0 (0%)	0 (0%)	0 (0%)			
4	0 (0%)	0 (0%)	0 (0%)	0 (0%)			
5	0 (0%)	0 (0%)	0 (0%)	0 (0%)			
6	1 (5.0%)	1 (5.0%)	0 (0%)	2 (3.3%)			
7	5 (25.0%)	4 (20.0%)	1 (5.0%)	10 (16.7%)			
8	14 (70.0%)	15 (75.0%)	19 (95.0%)	48 (80.0%)			
Left A							
1	0 (0%)	0 (0%)	0 (0%)	0 (0%)	W=10, p=0.036	W=190, p<0.001	W=171, p<0.001
2	0 (0%)	0 (0%)	0 (0%)	0 (0%)			
3	2 (10.0%)	0 (0%)	0 (0%)	2 (3.3%)			
4	11 (55.0%)	11 (55.0%)	0 (0%)	22 (36.7%)			
5	6 (30.0%)	8 (40.0%)	10 (50.0%)	24 (40.0%)			
6	1 (5.0%)	1 (5.0%)	8 (40.0%)	10 (16.7%)			
7	0 (0%)	0 (0%)	2 (10.0%)	2 (3.3%)			
8	0 (0%)	0 (0%)	0 (0%)	0 (0%)			
Left B							
1	0 (0%)	0 (0%)	0 (0%)	0 (0%)	W=32, p=0.021	W=171, p<0.001	W=129, p<0.001
2	0 (0%)	0 (0%)	0 (0%)	0 (0%)			
3	0 (0%)	0 (0%)	0 (0%)	0 (0%)			
4	0 (0%)	0 (0%)	0 (0%)	0 (0%)			
5	7 (35.0%)	5 (25.0%)	0 (0%)	12 (20.0%)			
6	9 (45.0%)	6 (30.0%)	6 (30.0%)	21 (35.0%)			
7	4 (20.0%)	9 (45.0%)	7 (35.0%)	20 (33.3%)			
8	0 (0%)	0 (0%)	7 (35.0%)	7 (11.7%)			
Left C							
1	0 (0%)	0 (0%)	0 (0%)	0 (0%)	W=10, p=0.036	W=78, p<0.001	W=49.5, p=0.007
2	0 (0%)	0 (0%)	0 (0%)	0 (0%)			
3	0 (0%)	0 (0%)	0 (0%)	0 (0%)			
4	0 (0%)	0 (0%)	0 (0%)	0 (0%)			
5	0 (0%)	0 (0%)	0 (0%)	0 (0%)			
6	2 (10.0%)	2 (10.0%)	0 (0%)	4 (6.7%)			
7	12 (60.0%)	8 (40.0%)	4 (20.0%)	24 (40.0%)			
8	6 (30.0%)	10 (50.0%)	16 (80.0%)	32 (53.3%)			
Left D							
1	0 (0%)	0 (0%)	0 (0%)	0 (0%)	NS	W=10, p=0.036	W=10, p=0.036
2	0 (0%)	0 (0%)	0 (0%)	0 (0%)			
3	0 (0%)	0 (0%)	0 (0%)	0 (0%)			
4	0 (0%)	0 (0%)	0 (0%)	0 (0%)			
5	0 (0%)	0 (0%)	0 (0%)	0 (0%)			
6	0 (0%)	0 (0%)	0 (0%)	0 (0%)			
7	4 (20.0%)	4 (20.0%)	0 (0%)	8 (13.3%)			
8	16 (80.0%)	16 (80.0%)	20 (100%)	52 (86.7%)			

Statistical analysis (Wilcoxon signed-rank test) indicated that the increase observed in the scores for visit 3 for parameters RA, RB, RC, RD, LA, LB, LC, and LD was statistically significant when compared to either visit 1 or visit 2 (p-value<0.05).

Analysis of quality of sleep

Comparison of Sleep Questionnaire Scores Between Different Visits for Group A

Table 11 provides a summary of the number of people and their respective responses to different question scores during different visits for Group A. As evident from Table 11, during visits 2 and 3, the percentage of individuals choosing response “A” for questions Q1, Q2, Q4, and Q5 increased significantly when compared to visit 1. The response “A” to these questions indicates the highest quality of life. For Q3, the number of individuals choosing response “D” increases significantly during visits 2 and 3 when compared to visit 1. Analysis of these results (graded responses to four questions) using the Wilcoxon signed-rank test indicated that all comparisons showed a p-value<0.05, indicating statistically significant differences.

**Table 11:** Sleep questionnaire scores of subjects in Group A across 3 visits Group A received MaQxan®-5:1 (lutein and zeaxanthin complex-5:1) for complete 8 months with a wash-off period of 15 days after the first 4 months. Level of significance was determined using the Wilcoxon signed-rank test. Q2 and Q5 are not statistically significant for comparison between visit 2 to visit 1. Q5 is not statistically significant for comparison between visit 3 to visit 1. Q1, Q2, Q3 and Q5 are not statistically significant for comparison between visit 3 and visit 2.

Group A	Visit 1	Visit 2	Visit 3	Overall	Test statistics and p-value		
	(N=20)	(N=20)	(N=20)	(N=60)	Visit 2 versus Visit 1	Visit 3 versus Visit 1	Visit 3 versus Visit 2
Q1	How many hours you sleep in a day						
A (More than 8 hours)	4 (20.0%)	15 (75.0%)	16 (80.0%)	35 (58.3%)	W=0, p<0.001	W=0, p<0.001	NS
B (Between 6 to 8 hours)	12 (60.0%)	5 (25.0%)	4 (20.0%)	21 (35.0%)			
C (Between 4 to 6 hours)	3 (15.0%)	0 (0%)	0 (0%)	3 (5.0%)			
D (Less than 4 hours)	1 (5.0%)	0 (0%)	0 (0%)	1 (1.7%)			
Q2	How many times in a day you sleep						
A (Only at night)	11 (55.0%)	13 (65.0%)	15 (75.0%)	39 (65.0%)	NS	W=0, p=0.031	NS
B (Afternoon and night)	6 (30.0%)	5 (25.0%)	5 (25.0%)	16 (26.7%)			
C (In between work)	2 (10.0%)	2 (10.0%)	0 (0%)	4 (6.7%)			
D (When I get time)	1 (5.0%)	0 (0%)	0 (0%)	1 (1.7%)			
Q3	How many times you wake up during sleep						
A (More than 3 times)	0 (0.0%)	0 (0.0%)	0 (0.0%)	0 (0.0%)	W=36, p=0.01	W=45, p=0.005	NS
B (2 to 3 times)	4 (20.0%)	1 (5.0%)	1 (5.0%)	6 (10.0%)			
C (At least once)	10 (50.0%)	6 (30.0%)	6 (30.0%)	22 (36.7%)			
D (Full good sleep)	6 (30.0%)	13 (65.0%)	13 (65.0%)	32 (53.3%)			
Q4	How you feel after getting up from sleep						
A (Very relaxed)	1 (5.0%)	4 (20.0%)	9 (45.0%)	14 (23.3%)	W=4.5, p=0.041	W=5.5, p=0.006	W=0, p=0.02
B (Relaxed)	16 (80.0%)	15 (75.0%)	11 (55.0%)	42 (70.0%)			
C (Disturbed)	2 (10.0%)	1 (5.0%)	0 (0%)	3 (5.0%)			
D (Very disturbed)	1 (5.0%)	0 (0%)	0 (0%)	1 (1.7%)			
Q5	How your eyes feel after waking up from sleep						
A (Good)	15 (75.0%)	16 (80.0%)	17 (85.0%)	48 (80.0%)	NS	NS	NS
B (Sleepy)	3 (15.0%)	3 (15.0%)	3 (15.0%)	9 (15.0%)			
C (Dry)	2 (10.0%)	1 (5.0%)	0 (0%)	3 (5.0%)			
D (Irritating)	0 (0%)	0 (0%)	0 (0%)	0 (0%)			

Comparison of Sleep Questionnaire Scores Between Different Visits for Group B

Table 12 provides a summary of the number of people and their respective responses to different question scores during different visits for Group B. The highest quality of life was suggested by this response. This trend was maintained during visit 3 as well. The response “A” to these questions indicates the highest quality of life. For Q3, the number of individuals choosing response “D” increases significantly during visits 2 and 3 when compared to visit 1. When analyzed by using the Wilcoxon signed-rank test, all the comparisons between visit 2 vs. visit 1 and visit 3 vs. visit 1 showed statistical significance (p-value<0.05). For the comparison between visit 2 and visit 3, the responses for Q4 and Q5 showed statistical significance (p-value<0.05).

**Table 12:** Sleep questionnaire scores of subjects in Group B across 3 visits Group B received MaQxan®-5:1 (lutein and zeaxanthin complex-5:1) for the first 4 months, had a wash-off period of 15 days, and then received a placebo for the next 4 months. Level of significance was tested using the Wilcoxon signed-rank test. Q2 is not statistically significant for comparison between visit 2 to visit 1 and between visit 3 to visit 1. None of the questions were statistically significant between visit 3 and visit 2. NS=not significant

Group B	Visit 1	Visit 2	Visit 3	Overall	Test statistics and p-value		
	(N=20)	(N=20)	(N=20)	(N=60)	Visit 2 versus Visit 1	Visit 3 versus Visit 1	Visit 3 versus Visit 2
Q1	How many hours you sleep in a day						
A (More than 8 hours)	2 (10.0%)	10 (50.0%)	10 (50.0%)	22 (36.7%)	W=0, p=0.001	W=0, p=0.001	NS
B (Between 6 to 8 hours)	12 (60.0%)	10 (50.0%)	10 (50.0%)	32 (53.3%)			
C (Between 4 to 6 hours)	6 (30.0%)	0 (0%)	0 (0%)	6 (10.0%)			
D (Less than 4 hours)	0 (0%)	0 (0%)	0 (0%)	0 (0%)			
Q2	How many times in a day you sleep						
A (Only at night)	20 (100%)	20 (100%)	20 (100%)	60 (100%)	NS	NS	NS
B (Afternoon and night)	0 (0%)	0 (0%)	0 (0%)	0 (0%)			
C (In between work)	0 (0%)	0 (0%)	0 (0%)	0 (0%)			
D (When I get time)	0 (0%)	0 (0%)	0 (0%)	0 (0%)			
Q3	How many times do you wake up during sleep						
A (More than 3 times)	2 (10.0%)	0 (0%)	0 (0%)	2 (3.3%)	W=136, p<0.001	W=136, p<0.001	NS
B (2 to 3 times)	7 (35.0%)	2 (10.0%)	2 (10.0%)	11 (18.3%)			
C (At least once)	8 (40.0%)	5 (25.0%)	5 (25.0%)	18 (30.0%)			
D (Full good sleep)	3 (15.0%)	13 (65.0%)	13 (65.0%)	29 (48.3%)			
Q4	How do you feel after getting up from sleep						
A (Very relaxed)	1 (5.0%)	5 (25.0%)	6 (30.0%)	12 (20.0%)	W=0, p=0.006	W=0, p=0.003	NS
B (Relaxed)	14 (70.0%)	13 (65.0%)	12 (60.0%)	39 (65.0%)			
C (Disturbed)	4 (20.0%)	2 (10.0%)	2 (10.0%)	8 (13.3%)			
D (Very disturbed)	1 (5.0%)	0 (0%)	0 (0%)	1 (1.7%)			
Q5	How your eyes feel after waking up from sleep						
A (Good)	11 (55.0%)	16 (80.0%)	16 (80.0%)	43 (71.7%)	W=0, p=0.007	W=0, p=0.012	NS
B (Sleepy)	3 (15.0%)	3 (15.0%)	2 (10.0%)	8 (13.3%)			
C (Dry)	2 (10.0%)	1 (5.0%)	1 (5.0%)	4 (6.7%)			
D (Irritating)	4 (20.0%)	0 (0%)	1 (5.0%)	5 (8.3%)			

Comparison of Sleep Questionnaire Scores Between Different Visits for Group C

Table 13 provides a summary of the number of people and their respective responses to different question scores during different visits for Group C. The number of individuals choosing to respond “A” for questions Q1, Q2, Q4, and Q5 was not statistically significantly different between visit 2 and visit 1. Similarly, it was not significantly different for Q3. However, there was an increase in the number of individuals choosing the response “A” for Q1, Q4, and Q5 during visit 3 compared to visit 1 or visit 2. Except for Q2, all comparisons between visit 3 to visit 1 and visit 3 to visit 2 showed a p-value<0.05, indicating statistically significant differences (Wilcoxon signed-rank test). As it appears from Table 13, the quality of sleep improved for all the groups (Group A, Group B, and Group C) during the visits after the treatment with lutein and zeaxanthin complex-5:1.

**Table 13:** Sleep questionnaire scores of subjects in Group C across 3 visits Group C placebo for the first 4 months, had a wash-off period of 15 days, and then received the MaQxan®-5:1 (lutein and zeaxanthin complex-5:1) for the next 4 months. Level of significance tested using Wilcoxon Signed rank Test. Comparison between visit 2 to visit 1 was non-significant (NS).

Group C	Visit 1	Visit 2	Visit 3	Overall	Test statistics and p-value		
	(N=20)	(N=20)	(N=20)	(N=60)	Visit 2 versus Visit 1	Visit 3 versus Visit 1	Visit 3 versus Visit 2
Q1	How many hours you sleep in a day						
A (More than 8 hours)	2 (10.0%)	2 (10.0%)	13 (65.0%)	17 (28.3%)	NS	W=0, p<0.001	W=0, p<0.001
B (Between 6 to 8 hours)	11 (55.0%)	12 (60.0%)	6 (30.0%)	29 (48.3%)			
C (Between 4 to 6 hours)	6 (30.0%)	5 (25.0%)	1 (5.0%)	12 (20.0%)			
D (Less than 4 hours)	1 (5.0%)	1 (5.0%)	0 (0%)	2 (3.3%)			
Q2	How many times in a day you sleep						
A (Only at night)	20 (100%)	20 (100%)	20 (100%)	60 (100%)	NS	NS	NS
B (Afternoon and night)	0 (0%)	0 (0%)	0 (0%)	0 (0%)			
C (In between work)	0 (0%)	0 (0%)	0 (0%)	0 (0%)			
D (When I get time)	0 (0%)	0 (0%)	0 (0%)	0 (0%)			
Q3	How many times you wake up during sleep						
A (More than 3 times)	5 (25.0%)	5 (25.0%)	0 (0%)	10 (16.7%)	NS	W=153, p<0.001	W=153, p<0.001
B (2 to 3 times)	4 (20.0%)	4 (20.0%)	3 (15.0%)	11 (18.3%)			
C (At least once)	8 (40.0%)	8 (40.0%)	2 (10.0%)	18 (30.0%)			
D (Full good sleep)	3 (15.0%)	3 (15.0%)	15 (75.0%)	21 (35.0%)			
Q4	How you feel after getting up from sleep						
A (Very relaxed)	2 (10.0%)	2 (10.0%)	10 (50.0%)	14 (23.3%)	NS	W=7.5, p<0.001	W=7, p<0.001
B (Relaxed)	11 (55.0%)	12 (60.0%)	10 (50.0%)	33 (55.0%)			
C (Disturbed)	6 (30.0%)	5 (25.0%)	0 (0%)	11 (18.3%)			
D (Very disturbed)	1 (5.0%)	1 (5.0%)	0 (0%)	2 (3.3%)			
Q5	How your eyes feel after waking up from sleep						
A (Good)	3 (15.0%)	3 (15.0%)	12 (60.0%)	18 (30.0%)	NS	W=0, p<0.001	W=0, p<0.001
B (Sleepy)	8 (40.0%)	9 (45.0%)	6 (30.0%)	23 (38.3%)			
C (Dry)	4 (20.0%)	3 (15.0%)	2 (10.0%)	9 (15.0%)			
D (Irritating)	5 (25.0%)	5 (25.0%)	0 (0%)	10 (16.7%)			

Clinical Safety and Laboratory Evaluation 

Intraocular Pressure

Intraocular pressure (IOP) is the fluid pressure inside the eye, which is essential for maintaining the eye’s shape and proper optical functioning. It results from the production and drainage of aqueous humor, the clear fluid filling the space between the cornea and the lens. Normal intraocular pressure ranges from 10 to 21 mmHg (millimeters of mercury), and its measurement is crucial in diagnosing and managing glaucoma, a group of eye conditions that can lead to vision loss due to optic nerve damage, often associated with high intraocular pressure. Maintaining balanced intraocular pressure is vital for ocular health, as both elevated and significantly low pressures can lead to vision problems [1].

Group A showed a slight decrease in intraocular pressure from visit 1 to visit 3 in both eyes, with the right eye having a more consistent decrease. The standard deviation was relatively stable, indicating consistent variability within the group. The value was well within the normal range and not clinically significant. The fluctuations observed in Groups B and C might suggest variability in treatment adherence, or they could be due to natural diurnal variations. All subjects demonstrated safety with lutein and zeaxanthin complex-5:1, and there was no adverse event (AE) with respect to intraocular pressure.

Retinal Thickness

In normal humans, the thickness varies across different regions of the retina, with the thickest part being the fovea, which is the central pit in the macula that provides the sharpest vision. On average, the foveal thickness is about 240 micrometers. The surrounding macular region has a thickness of approximately 300 micrometers, while the peripheral retina is thinner, measuring around 100 micrometers. Optical coherence tomography (OCT), a non-invasive imaging test, can obtain these measurements by providing detailed cross-sectional images of the retina. Normal retinal thickness is essential for proper visual function, and deviations from the norm can indicate various retinal conditions or diseases [30].

Retinal thickness tends to decrease with age, with an average reduction of about 0.24 μm for every year of age. Age-related changes in the retina include reductions in the thickness of several layers, such as the ganglion cell layer (GCL), inner plexiform layer (IPL), outer nuclear layer (ONL), and photoreceptor (PR) layer. Interestingly, some studies have found an increase in the thickness of the outer plexiform layer (OPL) with age. These changes are part of the natural aging process and may contribute to the decline in visual capacities observed in older individuals. Gender differences have also been noted, with women generally having lower mean thicknesses in several retinal layers compared to men [10,23].

Group A showed a decrease in central retinal thickness from visit 1 to visit 2, followed by a return to near baseline at visit 3 in both eyes. The average retinal thickness slightly increased over the three visits. This suggested that the medication does not have a long-term thinning effect on the retina, which was a positive sign for safety. Group B demonstrated a decrease in central retinal thickness from visit 1 to visit 2, with a slight increase at visit 3 in the right eye and a more consistent decrease across visits in the left eye. The average retinal thickness increased and then decreased slightly, but remains within a close range. Group C has a pattern similar to Group A, with a decrease from visit 1 to visit 2 and then an increase at visit 3 in central retinal thickness. The average retinal thickness increased slightly over time. This pattern also suggested no significant long-term thinning effect on the retina. Overall, there were no drastic changes in retinal thickness.

These observations suggest there was no clinically significant difference in the retinal thickness of the subjects. This supports the clinical safety of lutein and zeaxanthin.

Assessment of Biochemical Parameters

Biochemical parameters of Group A between different visits

Table 14 provides the mean ± standard deviation for different parameters for Group A during visits 1, 2, and 3. All these parameters did not show statistically significant change during the visits (p-value>0.05). There was a clinically significant reduction in HbA1C value between visit 1 and visit 3 among subjects taking lutein in Group A [39].

**Table 14:** Mean ± standard deviation for different biochemical parameters for subjects in Group-A during visits 1, 2, and 3 Group A received MaQxan®-5:1 (lutein and zeaxanthin complex-5:1) for complete 8 months with a wash-off period of 15 days after the first 4 months. *Clinically significant change in HbA1c between visit 3 and visit 1. ALP: alkaline phosphatase, ALT: alanine transaminase, AST: aspartate aminotransferase, GGT: gamma-glutamyl transferase, TT3: total triiodothyronine, TT4: total thyroxine, TSH: thyroid-stimulating hormone

Visit	Reference range	Visit 1	Visit 2	Visit 3	F-value	p-value
HbA1c (%)	4.0-5.6	5.89 ± 0.67	5.63 ± 1.08	5.39 ± 1.22*	1.263	0.291
Serum creatinine (mg/dL)	0.74-1.35	0.95 ± 0.19	1.4 ± 1.58	1.0 ± 0.23	1.36	0.265
Urea (g/24h)	26-43	24.41 ± 4.27	23.94 ± 6.72	26.09 ± 4.89	0.219	0.804
Sodium (mEq/L)	135-145	140.85 ± 2.66	137.84 ± 17.12	139.3 ± 6.57	0.237	0.79
Potassium (mEq/L)	3.5-5.2	4.8 ± 2.34	4.16 ± 0.36	4.69 ± 0.79	1.91	0.158
Chloride (mEq/L)	96-106	103.1 ± 2.61	107.67 ± 25.78	108.9 ± 13.82	0.589	0.558
Uric acid (mg/dL)	4.0-8.5	4.38 ± 1.32	5.12 ± 3.79	28.73 ± 61.45	1.707	0.191
Bilirubin total (mg/dL)	0.3-1.0	0.55 ± 0.22	0.64 ± 0.61	0.44 ± 0.22	0.21	0.811
Bilirubin direct (mg/dL)	0.1-0.3	0.14 ± 0.05	0.15 ± 0.05	0.16 ± 0.08	2.178	0.123
Bilirubin indirect (mg/dL)	0.2-0.8	0.41 ± 0.19	0.42 ± 0.31	0.5 ± 0.23	0.615	0.544
ALP (IU/L)	44-147	90 ± 19.45	83.5 ± 18.29	89.6 ± 26.51	1.521	0.228
ALT (U/L)	7-55	35.6 ± 17.97	32.3 ± 14.72	38.45 ± 11.88	1.892	0.161
AST (U/L)	8-48	31.5 ± 18.3	33.04 ± 18.04	36.55 ± 10.82	0.065	0.937
GGT (U/L)	8-61	27.4 ± 7.34	31.75 ± 7.94	27.41 ± 13.15	0.026	0.974
TT3 (ng/dL)	60-180	126.28 ± 14.82	123.33 ± 18.4	119.77 ± 20.23	1.053	0.356
TT4 (µg/dL)	5.4-11.5	9.34 ± 1.01	9.16 ± 1.17	7.47 ± 3.16	1.327	0.274
TSH (mIU/L)	0.5-5.0	3.07 ± 1.86	4.94 ± 5.73	4.04 ± 3.54	1.188	0.313

Biochemical parameters of Group B between different visits

Table 15 provides the mean ± standard deviation for different parameters for Group B during visits 1, 2, and 3. All these parameters did not show a statistically significant change during the visits (p-value>0.05).

**Table 15:** Mean ± standard deviation for different biochemical parameters for subjects in Group B during visits 1, 2, and 3 Group B received MaQxan®-5:1 (lutein and zeaxanthin complex-5:1) for the first 4 months, had a wash-off period of 15 days, and then received a placebo for the next 4 months. ALP: alkaline phosphatase, ALT: alanine transaminase, AST: aspartate aminotransferase, GGT: gamma-glutamyl transferase, TT3: total triiodothyronine, TT4: total thyroxine, TSH: thyroid-stimulating hormone

Visit	Reference range	Visit 1	Visit 2	Visit 3	F-value	p-value
HbA1c (%)	4.0-5.6	5.87 ± 1.12	6.2 ± 1.54	5.86 ± 0.72	1.039	0.361
Serum creatinine (mg/dL)	0.74-1.35	1.1 ± 0.25	0.98 ± 0.22	3.42 ± 10.85	0.211	0.81
Urea (g/24h)	26-43	26.59 ± 6.42	25.86 ± 6.25	23.61 ± 7.35	0.816	0.447
Sodium (mEq/L)	135-145	140.2 ± 2.19	141.85 ± 2.87	141 ± 2.94	0.712	0.495
Potassium(mEq/L)	3.5-5.2	4.22 ± 0.32	4.84 ± 2.34	4.47 ± 0.45	0.492	0.614
Chloride (mEq/L)	96-106	103.05 ± 3.36	103.9 ± 2.85	103.8 ± 3.24	1.152	0.324
Uric acid (mg/dL)	4.0-8.5	3.92 ± 1.12	4.16 ± 1.35	4.3 ± 1.18	0.352	0.705
Bilirubin total (mg/dL)	0.3-1.0	0.52 ± 0.18	0.66 ± 0.38	0.52 ± 0.24	0.089	0.915
Bilirubin direct (mg/dL)	0.1-0.3	0.12 ± 0.04	0.17 ± 0.13	0.14 ± 0.05	0.08	0.923
Bilirubin indirect (mg/dL)	0.2-0.8	0.4 ± 0.16	0.5 ± 0.27	0.38 ± 0.21	0.16	0.853
ALP (IU/L)	44-147	95.3 ± 26.59	86.45 ± 28.24	77.35 ± 20.98	0.186	0.83
ALT (U/L)	7-55	37.95 ± 19.32	41.45 ± 39.95	36.2 ± 14.04	0.895	0.414
AST (U/L)	8-48	28.1 ± 11.04	34.55 ± 20.14	35.3 ± 22.38	0.995	0.376
GGT (U/L)	8-61	29.35 ± 13.43	31.65 ± 14.18	29.8 ± 14.7	0.008	0.992
TT3 (ng/dL)	60-180	125.85 ± 17.21	118.8 ± 20.59	123.48 ± 19.04	1.691	0.194
TT4 (µg/dL)	5.4-11.5	8.72 ± 1.08	8.74 ± 1.17	8.71 ± 1.34	0.745	0.48
TSH (mIU/L)	0.5-5.0	5.15 ± 6.6	3.14 ± 3.68	3.42 ± 3.66	0.731	0.486

Biochemical parameters of Group C between different visits

Table 16 provides the mean ± standard deviation for different parameters for Group C during visits 1, 2, and 3. Most of these parameters did not show statistically significant change during the visits (p-value>0.05).

**Table 16:** Mean ± standard deviation for different biochemical parameters for subjects in Group C during visits 1, 2, and 3 Group C placebo for the first 4 months, had a wash-off period of 15 days, and then received the MaQxan®-5:1 (lutein and zeaxanthin complex-5:1) for the next 4 months. ALP: alkaline phosphatase, ALT: alanine transaminase, AST: aspartate aminotransferase, GGT: gamma-glutamyl transferase, TT3: total triiodothyronine, TT4: total thyroxine, TSH: thyroid-stimulating hormone

Visit	Reference range	Visit 1	Visit 2	Visit 3	F-value	p-value
HbA1c (%)	4.0-5.6	6.06 ± 1.44	5.96 ± 1.21	6.2 ± 1.54	1.225	0.302
Serum creatinine (mg/dL)	0.74-1.35	1.06 ± 0.24	0.96 ± 0.21	0.99 ± 0.18	0.065	0.937
Urea (g/24h)	26-43	26.16 ± 4.94	25.32 ± 5.82	24.64 ± 4.79	0.566	0.571
Sodium (mEq/L)	135-145	141.55 ± 2.74	141.7 ± 2.54	141.8 ± 2.73	0.229	0.796
Potassium(mEq/L)	3.5-5.2	4.64 ± 0.8	4.3 ± 0.38	4.35 ± 0.34	0.434	0.65
Chloride (mEq/L)	96-106	103.1 ± 3.24	103.3 ± 2.72	102 ± 3.34	0.827	0.443
Uric acid (mg/dL)	4.0-8.5	4.42 ± 1.5	4.45 ± 1.47	3.76 ± 1.2	1.342	0.27
Bilirubin total (mg/dL)	0.3-1.0	0.74 ± 0.38	0.56 ± 0.28	0.54 ± 0.35	0.133	0.876
Bilirubin direct (mg/dL)	0.1-0.3	0.17 ± 0.07	0.34 ± 0.32	0.25 ± 0.33	3.116	0.055
Bilirubin indirect (mg/dL)	0.2-0.8	0.57 ± 0.33	0.44 ± 0.26	0.41 ± 0.29	0.165	0.848
ALP (IU/L)	44-147	84.95 ± 25.34	59.65 ± 27.39	77.7 ± 29.4	2.369	0.329
ALT (U/L)	7-55	43.15 ± 21.41	30.8 ± 18.23	30.4 ± 20.51	1.831	0.71
AST (U/L)	8-48	37.7 ± 37.51	30.35 ± 10.19	30.15 ± 18.18	1.282	0.286
GGT (U/L)	8-61	35.85 ± 31.04	30.85 ± 13.22	31.35 ± 15.7	2.951	0.061
TT3 (ng/dL)	60-180	117.74 ± 14.75	124.22 ± 15.32	124.7 ± 17.59	0.661	0.52
TT4 (µg/dL)	5.4-11.5	9.11 ± 1.3	6.67 ± 4.65	7.18 ± 3.09	2.877	0.29
TSH (mIU/L)	0.5-5.0	1.97 ± 0.80	2.61 ± 0.73	2.16 ± 3.79	2.222	0.12

Reporting of adverse events

Two subjects under Group A reported flatulence as a mild adverse event. However, both these subjects continued and completed the trial duration. No other adverse effects or severe adverse effects were observed in this trial.

## Discussion

Macular carotenoids, lutein, and zeaxanthin are essential for maintaining eye health and protecting against diseases like AMD [1,2,40,41]. Human pharmacokinetic studies show that consuming lutein and zeaxanthin through diet or supplementation increases their levels in the blood, which enhances their deposition in the macula. However, due to poor intestinal absorption and stability, lutein and zeaxanthin in their natural form have low bioavailability after oral intake, leading to variable plasma concentrations [42].

Age is positively correlated with macular pigment (MP) levels, even within the narrow age range of the study's subjects. While some studies show a decrease in MPOD with age [29,30,43,44], others report no change or even an increase [45,46]. A higher dose of lutein and zeaxanthin leads to increased serum concentrations, although factors like intestinal absorption, metabolism, and serum clearance also affect circulating levels [47].

Increased MPOD levels, which offer better visual protection and performance, are linked to higher circulating lutein and zeaxanthin levels. Evidence suggests that supplements like the AREDS2 formulation (10 mg lutein and 2 mg zeaxanthin daily) may stabilize or improve best-corrected visual acuity in patients at risk of advanced AMD. There is some evidence that subjects receiving AREDS2-type supplements may have stabilization and improvement of best-corrected visual acuity. The AREDS2 formulation, which contains 10 mg lutein/day and 2 mg zeaxanthin/day, is currently the standard of care for slowing the progression of AMD from the intermediate to late stages [24,48]. In this study, supplementation with lutein and zeaxanthin complex-5:1 significantly increased MPOD levels, suggesting that it likely raised circulating lutein and zeaxanthin levels, although serum analysis was not conducted.

Other studies [36,49] showed that increased plasma circulation levels of lutein and zeaxanthin increased MPOD [43], and provided health advantages such as improved disability glare performance, improved contrast sensitivity, and photostress recovery [50,51]. The antioxidant and anti-inflammatory properties of lutein and zeaxanthin support the retina and the choroidal blood vessels that provide blood to the macular part of the retina, protecting it from blue light [13]. Improved visual acuity, sensitivity, glare performance [13,42,50,51], and reduced AMD risk [46,53] are a result of increased MPOD in the macula [44,52]. Lutein and zeaxanthin at a 5:1 dietary ratio have been thoroughly investigated in numerous clinical trials at a dosage of 10 mg lutein and 2 mg zeaxanthin, and it has been demonstrated to provide optimal eye-related health benefits while being safe [54].

This study has several strengths, including a relatively large sample size, a randomized, double-masked, placebo-controlled design, and an objective MPOD measurement method that minimizes observer bias. Results showed that the average MPOD-RIGHT and LEFT in the treatment group was approximately two times higher than the placebo group. These differences were statistically significant (p<0.05, paired t-test). In Group B it was observed that MPOD values were consistent between visit-2 and visit-3. This may be due to the retention effect of supplementation.

In Group C, MPOD values between visit-1 and visit-2 were not significantly different, likely due to the placebo treatment. However, MPOD levels increased significantly between visit-1 and visit-3, as well as between visit-2 and visit-3, indicating the effects of supplementation between those visits.

The contrast sensitivity scores for all parameters except LB were significantly higher in the treated group compared to the untreated group, as measured by the Wilcoxon signed rank test (p value<0.05). The contrast sensitivity scores for RA, RB, RC, RD, LC, and LD were not significantly different between the treatment and placebo groups. However, for parameters LA and LB, it was higher in the treatment group (as measured by the Wilcoxon signed rank test, p value<0.05).

In the case of the quality of sleep questionnaire, the frequency distribution indicates that the treated group reported higher quality of sleep scores. Except for Q2, for all other questions, these observations were statistically significant as measured by the Wilcoxon signed rank test (p value<0.05).

Based on the results observed during the study period, it can be stated that supplementing with lutein and zeaxanthin complex-5:1 may significantly improve their oral absorption, leading to increased serum levels, thus helping increase MPOD and other parameters, such as contrast sensitivity and quality of sleep.

Although this study provides important insights into the positive effects of lutein and zeaxanthin supplementation, it is not without limitations. The relatively small sample size may affect the generalizability of the findings to a larger population. Additionally, the short duration of the study limits our ability to assess the long-term effects of intervention. Furthermore, the study was conducted among specific population, which may limit its applicability to other demographic groups. Despite these limitations, the study's rigorous design and use of validated measures strengthen its reliability. Future research should aim to include larger, more diverse samples and evaluate outcomes over extended periods to build on these findings.

## Conclusions

The significant visual challenges associated with the rapid increase in near-field screen usage are a growing concern in the digital age, where avoiding screen exposure is nearly impossible. Blue light emitted from digital screens directly affects macular pigment levels and sleep quality. The findings of this study strongly support the regular use of lutein and zeaxanthin complex-5:1 supplementation to enhance MPOD levels. This supplementation not only improves macular pigment levels but also enhances visual acuity, contrast sensitivity, and sleep quality.

These results support the benefits of lutein and zeaxanthin in improving MPOD and promoting overall eye health. Furthermore, the study recorded no adverse effects on visual functions or other biochemical parameters, confirming the safety of prolonged lutein and zeaxanthin supplementation. This study provides a foundation for developing therapeutic regimens incorporatinglutein and zeaxanthin to maintain optimal eye health in the digital era, allowing individuals to sustain their routines without compromising visual well-being.
